# Causal theory error in college students’ understanding of science studies

**DOI:** 10.1186/s41235-021-00347-5

**Published:** 2022-01-12

**Authors:** Colleen M. Seifert, Michael Harrington, Audrey L. Michal, Priti Shah

**Affiliations:** Department of Psychology, University of Michigan, 530 Church St, Ann Arbor, MI 48109 USA

**Keywords:** Theory-evidence coordination, Correlation and causation, Causal inference, Science education, Science communication

## Abstract

When reasoning about science studies, people often make *causal theory errors* by inferring or accepting a causal claim based on correlational evidence. While humans naturally think in terms of causal relationships, reasoning about science findings requires understanding how evidence supports—or fails to support—a causal claim. This study investigated college students’ thinking about causal claims presented in brief media reports describing behavioral science findings. How do science students reason about causal claims from correlational evidence? And can their reasoning be improved through instruction clarifying the nature of causal theory error? We examined these questions through a series of written reasoning exercises given to advanced college students over three weeks within a psychology methods course. In a pretest session, students critiqued study quality and support for a causal claim from a brief media report  suggesting an association between two variables. Then, they created diagrams depicting possible alternative causal theories. At the beginning of the second session, an instructional intervention introduced students to an extended example of a causal theory error through guided questions about possible alternative causes. Then, they completed the same two tasks with new science reports immediately and again 1 week later. The results show students’ reasoning included fewer causal theory errors after the intervention, and this improvement was maintained a week later. Our findings suggest that interventions aimed at addressing reasoning about causal claims in correlational studies are needed even for advanced science students, and that training on considering alternative causal theories may be successful in reducing casual theory error.

## Significance statement

*Causal theory error—*defined as making a causal claim based on correlational evidence—is ubiquitous in science publications, classrooms, and media reports of scientific findings. Previous studies have documented causal theory error as occurring on a par with correct causal conclusions. However, no previous studies have identified effective interventions to improve causal reasoning about correlational findings. This study examines an example-based intervention to guide students in reasoning about plausible alternative causal theories consistent with the correlational evidence. Following the intervention, advanced college students in a research methods course offered more critiques of causal claims and generated more alternative theories, and then maintained these gains a week later. Our results suggest that causal theory error is common even in college science courses, but interventions focusing on considering alternative theories to a presented causal claim may be helpful. Because behavioral science communications in the media are increasingly available, helping people improve their ability to assess whether evidence from science studies support making changes in behavior, thinking, and policies is an important contribution to science education.

## Introduction

Causal claims from research studies shared on social media often exceed the strength of scientific evidence (Haber et al., 2018). This occurs in journal articles as well; for example, a recent study in *Proceedings of the National Academy of Sciences* reported a statistical association between higher levels of optimism and longer life spans, concluding, “…optimism serves as a psychological resource that promotes health and longevity” (Lee et al., 2019, p. 18,360). Given evidence of association, people (including scientists) readily make a mental leap to infer a causal relationship. This error in reasoning—*cum hoc ergo propter hoc* (“with this, therefore because of this”)—occurs when two coinciding events are assumed to be related through cause and effect. A press release for the article above offered, “If you’re happy and you know it… you may live longer” (Topor, 2019). But will you? What critical thinking is needed to assess this claim?

## Defining causal theory error

The tendency to infer causation from correlation—referred to here as *causal theory error*—is arguably the most ubiquitous and wide-ranging error found in science literature (Bleske-Rechek et al., 2018; Kida, 2006; Reinhart et al., 2013; Schellenberg, 2020; Stanovich, 2009), classrooms (Kuhn, 2012; Mueller & Coon, 2013; Sloman & Lagando, 2003), and media reports (Adams et al., 2019; Bleske-Rechek et al., 2018; Sumner et al., 2014). While science pedagogy informs us that, “correlation does not imply causation” (Stanovich, 2010), the human cognitive system is “built to see causation as governing how events unfold” (Sloman & Lagnado, 2015, p. 32); consequently, people interpret almost all events through causal relationships (Corrigan & Denton, 1996; Hastie, 2015; Tversky & Kahneman, 1977; Sloman, 2005), and act upon them with unwarranted certainty (Kuhn, 2012). Claims about causal relationships from correlational findings are used to guide decisions about health, behavior, and public policy (Bott et al., 2019; Huggins-Manley et al., 2021; Kolstø et al., 2006; Lewandowsky et al., 2012). Increasingly, science media reports promote causal claims while omitting much of the information needed to evaluate the scientific evidence (Zimmerman et al., 2001), and editors may modify media report headlines to make such reports more eye-catching (Jensen, 2008). As a result, causal theory error is propagated in a third of press releases and over 80% of their associated media stories (Sumner et al., 2014).

The ability to reason about causal relationships is fundamental to science education (Blalock, 1987; Jenkins, 1994; Kuhn, 1993, 2005; Miller, 1996; Ryder, 2001), and U.S. standards aim to teach students to critically reason about covariation starting in middle school (Lehrer & Schauble, 2006; National Science Education Standards, 1996; Next Generation Science Standards, 2013). To infer causation, a controlled experiment involving direct manipulation and random assignment to treatment and control conditions is the “gold standard” (Hatfield et al., 2006; Koch & Wüstemann, 2014; Reis & Judd, 2000; Sullivan, 2011). However, for many research questions, collecting experimental evidence from human subjects is expensive, impractical, or unethical (Bensley et al., 2010; Stanovich, 2010), so causal conclusions are sometimes drawn without experimental evidence (Yavchitz et al., 2012). A prominent example is the causal link between cigarette smoking and causes of death: “For reasons discussed, we are of the opinion that the associations found between regular cigarette smoking and death … reflect cause and effect relationships” (Hammond & Horn, 1954, p. 1328). Nonexperimental evidence (e.g., longitudinal data)—along with, importantly, the lack of plausible alternative explanations—sometimes leads scientists to *accept* a causal claim supported only by correlational evidence (Bleske-Rechek et al., 2018; Marinescu et al., 2018; Pearl & Mackenzie, 2018; Reinhart et al., 2013; Schellenberg, 2020). With no hard-and-fast rules regarding the evidence necessary  to conclude causation, the *qualities* of the evidence offered are evaluated to determine the appropriateness of causal claims from science studies (Kuhn, 2012; Morling, 2014; Picardi & Masick, 2013; Steffens et al., 2014; Sloman, 2005).

## Theory-evidence coordination in causal reasoning

To reason about claims from scientific evidence, Kuhn and colleagues propose a broad-level process of *theory-evidence coordination* (Kuhn, 1993; Kuhn et al., 1988), where people use varied strategies to interpret the implications of evidence and align it with their theories (Kuhn & Dean, 2004; Kuhn et al., 1995). In one study, people read a scenario about a study examining school features affecting students' achievement (Kuhn et al, 1995, p. 29). Adults examined presented  data points and detected simple covariation to (correctly) conclude that, “having a teacher assistant causes higher student achievement” (Kuhn et al, 1995). Kuhn (2012) suggests theory-evidence coordination includes generating some conception as to why an association “makes sense” by drawing on background knowledge to make inferences, posit mechanisms, and consider the plausibility of a causal conclusion (Kuhn et al., 2008). Assessments of causal relationship are biased in the direction of prior expectations and beliefs (e.g., Billman et al., 1992; Fugelsang & Thompson, 2000, 2003; Wright & Murphy, 1984). In general, people focus on how, “…the evidence demonstrates (or at least illustrates) the theory’s correctness, rather than that the theory remains likely to be correct in spite of the evidence” (Kuhn & Dean, 2004, p. 273).

However, in causal theory error, the process of theory-evidence coordination may require *less* attention to evidence. Typically, media reports of science studies present a summary of associational evidence that corroborates a presented theoretical causal claim (Adams et al., 2019; Bleske-Rechek et al., 2018; Mueller, 2020; Sumner et al., 2014). Because the evidence is often summarized as a probabilistic association (for example, a statistical correlation between two variables), gathering more evidence from instances cannot confirm or disconfirm the causal claim (unlike reasoning about instances, as in Kuhn et al., 1995). Instead, the reasoner must evaluate a presented causal theory by considering *alternative* theories also corresponding with the evidence, disproving internal validity (that the presented causal claim is the only possibility). In the student achievement example (Kuhn et al., 1995), a causal theory error occurs by failing to consider that the theory—”having a teacher assistant causes higher student achievement”—is not the *only* causal theory consistent with the evidence; for example, they may co-occur in schools due to a third variable, such as school funding. In the case of causal theory error, the presented causal claim is *consistent* with the evidence; however, the non-experimental evidence cannot support *only* the presented causal claim. Rather than an error in coordinating theory and evidence (Kuhn, 1993; Kuhn et al., 1995), we propose that causal theory error  arises from failure to examine the *uniqueness* of a given causal theory .

Theory-evidence coordination is often motivated by contextually rich, elaborate, and familiar content along with existing beliefs, but these may also interfere with proficient scientific thinking (Billman et al., 1992; Fugelsang & Thompson, 2000, 2003; Halpern, 1998; Koehler, 1993; Kuhn et al., 1995; Wright & Murphy, 1984). Everyday contexts more readily give rise to personal beliefs, prior experiences, and emotional responses (Shah et al., 2017); when congruent or desirable with a presented claim, these may increase plausibility and judgments of quality (Michal et al., 2021; Shah et al., 2017). Analytic, critical thinking may occur more readily when information *conflicts* with existing beliefs (Evans & Curtis-Holmes, 2005; Evans, 2003a, b; Klaczynski, 2000; Kunda, 1990; Nickerson, 1998; Sá et al., 1999; Sinatra et al., 2014). Consequently, learning to recognize causal theory error may require guiding students in “reasoning through” their own related beliefs to identify how they may align—and not align—with the evidence given. Our approach in this study takes advantage of students’ ability to access familiar contexts and world knowledge to create their own alternative causal theories.

## Prevalence of causal theory error

Prior research on causal reasoning has typically examined the formation of causal inferences drawn from observing feature co-occurrence in examples (e.g., Kuhn et al., 1995; Cheng, 1997; Fugelsang & Thompson, 2000, 2003; Griffiths & Tenenbaum, 2005; see also Sloman & Lagnado, 2015). Fewer studies have asked people to evaluate causal claims based on summary evidence (such as stating there is a correlation between two variables). A study by Steffens and colleagues (2014) asked college students to judge the appropriateness of claims from evidence in health science reports. Students were more likely to reject causal claims (e.g., ‘School lunches cause childhood obesity”) from correlational than from experimental studies. However, each report in the study explicitly stated, “Random assignment is a gold standard for experiments, because it rules out alternative explanations. This procedure [does, does not] allow us to rule out alternative explanations” (Steffens et al., 2014; p. 127). Given that each reported study was labelled as allowing (or not allowing) a causal claim (Steffens et al., 2014), these findings may overestimate people’s ability to avoid causal theory errors.

A field study recruiting people in restaurants to evaluate research reports provides evidence that causal theory errors are quite common. In this study (Bleske-Rechek et al., 2015), people read a research scenario linking two variables (e.g., playing video games and aggressive playground behavior) set in either experimental (random assignment to groups) or non-experimental (survey) designs. Then, they selected appropriate inferences among statements including causal links (e.g., “Video games cause more aggression”), reversed causal links (“Aggression causes more video game playing”), and associations (e.g., “Boys who spend more time playing video games tend to be more aggressive”). Across three scenarios, 63% of people drew causal inferences from non-experimental data, just as often as from experimental findings. Further, people were more likely to infer directions of causality that coincided with common-sense notions (e.g., playing games leads to more aggression rather than the reverse) (Bleske-Rechek et al., 2015).

Another study by Xiong et al. (2020) found similarly high rates of endorsing causal claims from correlational evidence. With a crowdsourced sample, descriptions of evidence were presented in text format, such as, "When students eat breakfast very often (more than 4 times a week), their GPA is around 3.5; while when students eat breakfast not very often (less than four times a week), their GPA is around 3.0,” or with added bar graphs, line graphs, or scatterplots (Xiong, et al., 2020, p. 853). Causal claims (e.g., “If students were to eat breakfast more often, they would have higher GPAs”) were endorsed by 67–76% of adults compared to 85% (on average) for correlational claims (“Students who more often eat breakfast tend to have higher GPA”) (Xiong, et al., 2020, p. 858). Finally, Adams et al. (2017) suggested people do not consistently distinguish among descriptions of “moderate” causal relationships, such as, “might cause,” and “associated with,” even though the wording establishes a logical distinction between causation and association. These studies demonstrate that causal theory error in interpreting science findings is pervasive, with most people taking away an inappropriate causal conclusion from associational evidence (Zweig & Devoto, 2015).

## Causal theory error in science students

However, compared to the general public, those with college backgrounds (who on average have more science education) have been shown to have better science evaluation skills (Amsel et al., 2008; Huber & Kuncel, 2015; Kosonen & Winne, 1995; Norcross et al., 1993). College students make better causal judgments when reasoning about science, including taking in given information, reasoning through to a conclusion, and tracking what they know and don’t know from it (Koehler, 1993; Kuhn, 2012). Might science literacy (Miller, 1996) help college students be more successful in avoiding causal theory error? Norris et al. (2003) found that only about a third of college students can correctly distinguish between causal and correlational statements in general science texts. And teaching scientific content alone may not improve scientific reasoning, as in cross-cultural studies showing Chinese students outperform U.S. students on measures of science content but perform equally on measures of scientific reasoning (Bao et al., 2009; Crowell & Schunn, 2016). College-level science classes (as many as eight) fail to predict performance on everyday reasoning tasks compared to high school students (Norris & Phillips, 1994; Norris et al., 2003). Despite more science education, college students still struggle to accurately judge whether causal inferences are warranted (Norris et al., 2003; Rodriguez et al., 2016a, b).

Because media reports typically describe behavioral studies (e.g., Bleske-Rechek et al., 2018; Haber et al., 2018), psychology students may fare better (Hall & Seery, 2006). Green and Hood (2013) suggest psychology students’ epistemological beliefs benefit from an emphasis on critical thinking, research methods, and integrating knowledge from multiple theories. Hofer (2000) found that first year psychology students believe knowledge is less certain and more changing in psychology than in science more generally. And Renken and colleagues (2015) found that psychology-specific epistemological beliefs, such as the subjective nature of knowledge, influence students' academic outcomes. Psychology exposes students to both correlational and experimental studies, fostering distinctions about the strength of empirical evidence (Morling, 2014; Reinhart et al., 2013; Stanovich, 2009). Mueller and Coon (2013) found students in an introductory psychology class interpreted correlational findings with just 28% error on average, improving to just 7% error at the end of the term. To consider causal theory error among psychology students, we recruited a convenience sample of advanced undergraduate majors in a research methods course. These students may be more prepared to evaluate causal claims in behavioral studies reported in the media.

## Correcting causal theory error

To attempt to remedy causal theory error, the present study investigates whether students’ reasoning about causal claims in science studies can be improved through an educational intervention. In a previous classroom study, Mueller and Coon (2013) introduced a special curriculum over a term in an introductory psychology course. By emphasizing how to interpret correlational findings, the rate of causal theory error decreased by 21%. Our study used a similar pre/post design to assess base rates of causal theory error and determine the impact of a single, short instructional intervention. Our intervention was based on guidelines from science learning studies identifying example-based instruction (Shafto et al., 2014; Van Gog & Rummel, 2010) and self-explanation (Chi et al., 1989). Renkl and colleagues found examples improve learning by promoting self-explanations of concepts through spontaneous, prompted, and trained strategies (Renkl et al., 1998; Stark et al., 2002, 2011). Even *incorrect* examples can be beneficial in learning to avoid errors (Durkin & Rittle-Johnson, 2012; Siegler & Chen, 2008). Based on evidence that explicit description of the error within an example facilitates learning (Große & Renkl, 2007), our intervention presented a causal theory error made in an example and explained why the specific causal inference was not warranted given the evidence (Stark et al., 2011).

Following Berthold and Renkl’s paradigm (2009, 2010), our intervention incorporated working through an extended example using world knowledge (Kuhn, 2012). To facilitate drawing on their own past experiences, the example study selected was relevant for recent high school graduates likely to have pre-existing beliefs and attitudes (both pro and con) toward the presented causal claim (Bleske-Rechek et al., 2015; Michal et al., 2021). Easily accessible world knowledge related to the study findings may assist students in identifying how independent thinking *outside* of the presented information can inform their assessment of causal claims. We encouraged students to think on their own about possible causal theories by going “beyond the given information” (Bruner, 1957). Open-ended prompts asked students to think through what would happen in the absence of the stated cause, whether a causal mechanism is evident, and whether the study groups differed in some way. Then, students generated their own alternative causal theories, including simple cause-chain and reverse cause-chain models, and potential third variables causing both (common causes) (Pearl, 1995, 2000; Shah et al., 2017).

To support students in identifying alternative causal theories, our intervention included visualizing causal models through the creation of diagrams. Creating diagrams after reading scientific material has been linked with better understanding of causal and dynamic relationships (Ainsworth & Loizou, 2003; Bobek & Tversky, 2016; Gobert & Clement, 1999). Students viewing diagrams generate more self-explanations (Ainsworth & Loizou, 2003), and training students to construct their own diagrams (“drawing to learn”) may promote additional frames for understanding difficult concepts (Ainsworth & Scheiter, 2021). Diagramming causal relationships may help students identify causal theories and assist them in considering alternatives. For simplicity, students diagrammed headlines from actual media reports making a causal claim; for example, ‘‘Sincere smiling promotes longevity” (Mueller, 2020). Assessing causal theory error with headlines minimizes extraneous study descriptions that may alter interpretations (Adams et al., 2017; Mueller & Coon, 2013).

We expected our short intervention to support students in learning to avoid causal theory error; specifically, we predicted that students would be more likely to notice when causal claims from associational evidence were unwarranted through increased consideration of alternative causal theories following the intervention.

## Method

### Participants

Students enrolled in a psychology research methods course at a large midwestern U.S. university were invited to participate in the study over three consecutive weeks during lecture sessions. The students enrolled during their third or fourth year of college study after completing introductory and advanced psychology courses and a prerequisite in statistics. The three study sessions (*pretest, intervention,* and *post-test*) occurred in weeks 3–5 of the 14-week course, prior to any instruction or readings on correlational or experimental methodology. Of 240 students enrolled, 97 (40%) completed the voluntary questionnaires for the study in all three sessions and were included in the analyses.

### Materials

#### Intervention

The text-based intervention explained how to perform theory-evidence coordination when presented with a causal claim through an extended example (see Appendix 1). The example described an Educational Testing Service (ETS) study reporting that 84% of top-tier workers (receiving the highest pay) had taken Algebra 2 classes in high school. Based on this evidence, legislatures in 20 states raised high school graduation requirements to include Algebra 2 (Carnevale et al., 2009). The study’s lead author, Anthony Carnevale, acknowledged this as a causal theory error, noting, “The causal relationship is very, very weak. Most people don’t use Algebra 2 in college, let alone in real life. The state governments need to be careful with this” (Whoriskey, 2011).

The intervention presented a short summary of the study followed by a series of ten questions in a worksheet format. The first two questions addressed the evidence for the causal claim and endorsement of the causal theory error, followed by five questions to prompt thinking about alternative explanations for the observed association, including reasoning by considering counterfactuals, possible causal mechanisms, equality of groups, self-selection bias, and potential third variables. For the final three questions, students were shown causal diagrams to assess their endorsement of the causal claim, the direction of causation, and potential third variables. The intervention also explicitly described this as an error in reasoning and ended with advice about the need to consider alternative causes when causal claims are made from associational evidence.

#### Dependent measures

**Fig. 1:**
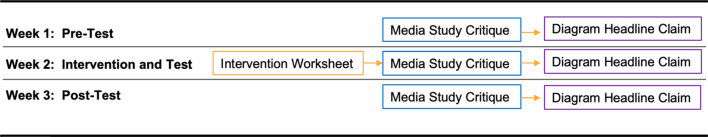
Graphical depiction of questionnaire content for each session in the longitudinal study

To investigate both students’ understanding of theory-evidence coordination in evaluating a study and their ability to generate alternative theories to the causal claim, we included two tasks—Study Critique and Diagram Generation—in each session (pretest, intervention, and post-test) as repeated measures (see Fig. 1). Each task included brief study descriptions paraphrased from actual science media reports (Mueller, 2020). To counter item-specific effects, three separate problem sets were generated pairing Critique and Diagram problems at random (see Appendix 2). The three problem sets were counterbalanced across students by assigning them at random to alternate forms so that each set of problems occurred equally frequently at each session.

In the Study Critique task, a short media story (between 100 and 150 words) was presented for evaluation; for example:

“…Researchers have recently become interested in whether listening to music actually helps students pay attention and learn new information. A recent study was conducted by researchers at a large midwestern university. Students (*n* = 450) were surveyed prior to final exams in several large, lecture-based courses, and asked whether they had listened to music while studying for the final. The students who listened to music had, on average, higher test scores than students who did not listen to music while studying. The research team concluded that students who want to do well on their exams should listen to music while studying.”

First, two rating items assessed the perceived quality of the study and its support for the claim with 5-point Likert scales, with “1” indicating low-quality and an unsupported claim, and “5” indicating high-quality and a supported claim. For both scales, higher scores indicate causal theory error (i.e., high quality study does support the causal claim). Next, an open-ended question asked for “a critical evaluation of the study’s method and claims, and what was good and bad about it” (see Appendix 3). Responses were qualitatively coded using four emergent themes: (1) pointing to causal theory error (correlation is not causation), (2) the study methodology (not experimental), (3) the identification of alternative theories such as third variables, and (4) additional studies are needed to support a causal claim. Each theme addresses a weakness in reasoning from the study’s correlational evidence to make a causal claim. Other issues mentioned infrequently and not scored as critiques included sample size, specialized populations, lack of doctor’s recommendation, and statistical analyses. Two authors worked independently to code a subset of the responses (10%), and the Cohen's Kappa value computed using SPSS was $\kappa$ = 0.916, indicating substantial agreement beyond chance on the Landis and Koch (1977) benchmarks. One author then scored the remaining responses. Higher scores on this *Critique Reasons* measure indicate greater ability to critique causal claims from associational data.

The Diagram Generation task included instructions with sample causal diagrams (Elwert, 2013) to illustrate how to depict relationships between variables (see Appendix 4); all students completed these correctly, indicating they understood the task. Then, a short “headline” causal claim was presented, such as, “Smiling increases longevity,” (Abel & Kruger, 2010) and students were asked to diagram possible relationships between the two variables. The *Alternative Theories* measure captured the number of distinct alternative causal relationships generated in diagrams, scored as a count of deductive categories including: (a) direct causal chain, (b) reverse direction chain, (c) common cause (a third variable causes both), and (d) multiple-step chains, with possible scores ranging from 0 to 4. Two authors worked independently to code a subset of the responses (10%), and the Cohen's Kappa value computed using SPSS was $\kappa$ = 0.973, indicating substantial agreement beyond chance on the Landis and Koch (1977) benchmarking scale. One author then scored the remainder of the responses. A higher *Alternative Theories* score reflects greater ability to generate different alternative causal explanations for an observed association.

### Procedure

The study took place during lecture sessions for an advanced psychology course over three weeks in the first third of the term. At the beginning of the lecture each week, students were asked to complete a study questionnaire. In the pretest session, students were randomly assigned to one of three alternate forms, and they received corresponding forms in the intervention and post-test sessions so that the specific science reports and headlines included in each student’s questionnaires were novel to them across sessions. Students were given 10 min to complete the intervention worksheet and 15 min to complete each questionnaire.

## Results

### Understanding the intervention

To determine how students understood the intervention and whether it was successful, we performed a qualitative analysis of open-ended responses. The example study presented summary data (percentages) of former students in high- and low-earning careers who took high school algebra and concluded that taking advanced algebra improves career earnings. Therefore, correct responses on the intervention reject this causal claim to avoid causal theory error. However, 66% of the students reported in their open-ended responses that they felt the study was convincing, and 63% endorsed the causal theory error as a “good decision” by legislators. To examine differences in students' reasoning, we divided the sample based on responses to these two initial questions.

The majority (*n* = 67) either endorsed the causal claim as convincing or agreed with the decision based on it (or both), and these students were more likely to cite “strong correlation” (25%; *χ*^2^ = 9.88, *p* = 0.0012, Φ = 0.171) and “foundational math is beneficial” (35%); *χ*^2^ = 8.278, *p* = 0.004, Φ = 0.164) as reasons. Students who rejected the causal claim (did not find the study convincing nor the decision supported; *n* = 33) showed signs of difficulty in articulating reasons for this judgment; however, 27% clearly identified causal theory error more often in their reasons (*χ*^2^ = 10.624, *p* = 0.001, Φ = 0.183). As Table 1 shows, the two groups' responses differ more initially (consistent with their judgments about the causal claim), but become more aligned on later questions.

**Table 1:** Intervention response frequencies for students rejecting versus endorsing causal claims

Open-ended response categories	Rejected (*n* = 29%)	Endorsed (71%)
*How does no requirement affect future opportunities?*		
Positively	24%	12%
^1^Negatively	7%	35%
Neither	62%	45%
Not Sure	7%	7%
*Why might taking algebra result in better jobs?*		
Looks good for jobs	44%	27%
Increases math skills	27%	30%
Learn creative problem solving	11%	19%
Shows college readiness	7%	14%
*In what ways might students taking algebra differ from students who don’t take it?*		
Highly motivated	20%	17%
Higher intelligence	16%	16%
Interested in math/STEM careers	26%	24%
Want to go to college	13%	12%
Parent/peer pressures	7%	11%
*Thinking back, why might students in the study have decided to take algebra?*		
Interested in learning math/careers	30%	22%
To get into college	4%	18%
Required for careers	14%	6%
*Thinking back, why might students in the study have decided not take algebra?*		
Too challenging/difficult	14%	22%
Not related to their field/career	11%	8%
No interest in learning it	6%	12%
*Alternative theories explain finding (smarter, college-bound, richer, better schools)?*		
^2^Endorsed 3 or 4	86%	60%
Endorsed two	7%	25%
Endorsed one or none	6%	14%
*Does this causal diagram make sense to you (yes/no responses)?*		
Taking algebra causes better jobs?	75%	66%
Better jobs cause taking algebra?	3%	7%
Being smart causes both?	90%	79%

Significant differences: ^1^*χ*^2^ = 7.756, *p* = .005, Φ = .14; ^2^*χ*^2^ = 6.229, *p* = .013, Φ = .125

On the final two questions, larger differences occur when considering possible alternative “third variables” responsible for both higher earnings and taking more algebra (“smarter,” “richer,” “better schools,” and “headed to college anyway”). More of these potential “third variables” were endorsed as plausible more often by students initially rejecting compared to accepting the causal claim. On a final question, in which three alternative causal diagrams (direct cause, reverse cause, third variable) were presented to illustrate possible causal relationships between Algebra 2 and top tier jobs, over 75% of both groups endorsed a third variable model. Based on these open-ended response measures, it appears most students understood the intervention, and most were able to reason about and accept alternatives to the stated causal claim.

### Analysis of causal theory error

We planned to conduct repeated measures ANOVAs with linear contrasts based on expected improvements on all three dependent variables (*Study Ratings, Critique Reasons*, and *Alternative Theories*) across *Sessions* (pre-test, immediately post-intervention, and delayed post-test). In this repeated-measures design, each student completed three questionnaires including the same six test problems appearing in differing orders. Approximately equal numbers of students completed each of three forms (*n* = 31, 34, 32). A repeated measures analysis with *Ordering* (green, white, or yellow) as a between-groups factor found no main effects, with no significant differences for *Ratings* (*F*(2, 94) = 0.996, *p* = 0.373), *Reasons* (*F*(2, 93) = 2.64, *p* = 0.08), or *Theories* (*F*(2, 93) = 1.84, *p* = 0.167). No further comparisons were made comparing order groups.

#### Study critiques: Ratings

The *Ratings* measures included two assessments: (1) the perceived quality of the study and (2) its support for the causal claim, with higher scores indicating causal theory error (i.e., a “high” quality study and “good” support for a causal claim). At pretest, ratings averaged above the midpoint of the 5-point rating scale for both quality and support, indicating that on average, students considered the study to be of good quality with moderate support for the causal claim. Immediately following the intervention, students ratings showed similar endorsments of study quality and support for a causal claim (see Table 2).

**Table 2:** Rating scale averages and standard deviations for pre-test, intervention, and post-test sessions

	Pre-test	Intervention	Post-test	*F*	*p*	*η*_p_^2^
Quality	3.15 (.894)	3.19 (.939)	2.86 (.890)	6.10	0.015*	.060
Support	3.37 (.961)	3.42 (.981)	3.10 (.941)	4.207	0.043*	.042

Linear contrasts with 1, 96 degrees of freedom, *p < .05

However, on the post-test one week later, ratings for both quality and support showed significant decreases. Planned linear contrasts showed a small improvement in rejecting a causal claim over the three sessions in both quality and support ratings, indicating less causal theory error at post-test. Using Tukey’s *LSD* test, the quality and support ratings in the post-test session were both significantly different from the pretest session (*p* = 0.015; 0.043) and intervention session (*p* = 0.014; 0.027), while the pretest and intervention means did not differ (*p* = 0.822; 0.739), respectively.

Both study quality and causal support ratings remained at pre-test levels after the intervention, but decreased in the final post-test session. About half (48%) of these advanced psychology students rated studies with correlational evidence as “supporting” a causal claim (above scale midpoint) on the pretest, with about the same number at the intervention session; however, at the post-test session, above mid-point support declined to 37%. This suggests students may view the ratings tasks as assessing consistency between theory and evidence (the *A* → *B* causal claim matches the study findings). For example, one student gave the study the highest quality rating and a midscale rating for support, but wrote in their open-ended critique that the study was, “bad—makes causation claims.” Another gave ratings of “4” on both scales but wrote that, “The study didn’t talk about other factors that could explain causal links, and as a correlation study, it can’t determine cause.” These responses suggest students may recognize a *correspondenc*e between the stated theory and the evidence in a study, yet reject the stated causal claim as *unique*. On the post-test, average ratings decreased, perhaps because students became more critical about what “support for a causal claim” entails. To avoid causal theory error, people must reason that the causal theory offered in the claim is not a unique explanation for the association, and this may not be reflected in ratings of study quality and support for causal claims.

### Study critiques: Reasons

**Fig. 2:**
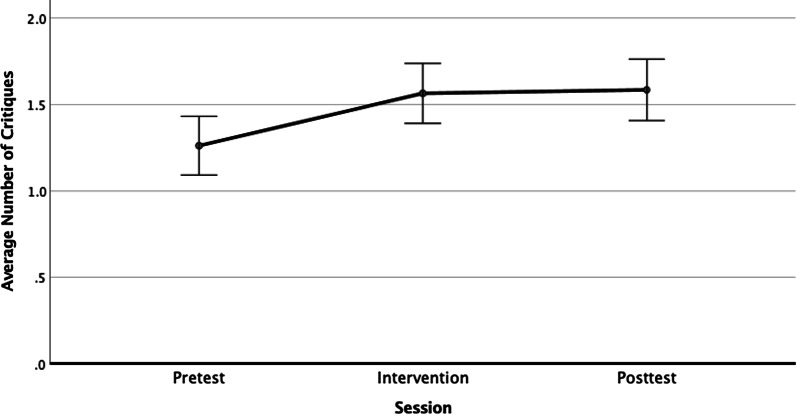
Average number of critique reasons in students’ open-ended responses across sessions. Error bars represent within-subjects standard error of the mean (Cousineau, 2005)

A second measure of causal reasoning was the number of *Critique Reasons* in the open-ended responses, scored as a count of coded themes related to causality (ranging from 0 to 4). These included stating that the study (a) was only correlational, (b) was not an experiment, (c) included other variables affecting the outcomes, and (d) required additional studies to support a causal claim. A planned linear contrast indicated a small improvement in *Critiques* scores after the pretest session, *F*(1, 94) = 9.318. *p* < 0.003, *η*_p_^2^ = 0.090, with significant improvement from pretest (*M* = 1.25; SD = 0.854) to intervention (*M* = 1.56, SD = 0.841) and post-test (*M* = 1.58, SD = 0.875). The intervention and post-test means were not different by Tukey’s *LSD* test (*p* = 0.769), but both differed from the pretest (intervention *p* = 0.009, post-test *p* = 0.003). The percentage of students articulating more than one correct reason to reject a causal claim increased from 31% on the pre-test to 47% immediately after the intervention, and this improvement was maintained on the post-test one week later, as shown in Fig. 2.

When considering “what’s good, and what’s bad” about the study, students were more successful in identifying causal theory error following the intervention, and they maintained this gain at the post-test. For example, in response to the claim that “controlling mothers cause kids to overeat,” one student wrote, “There may be a correlation between the 2 variables, but that does not necessarily mean that one causes the other. There could be another answer to why kids have more body fat.” Another wrote, “This is just a correlation, not a causal study. There could be another variable that might be playing a role. Maybe more controlling mothers make their kids study all day and don’t let them play, which could lead to fat buildup. So, the controlling mother causes lack of exercise, which leads to fat kids.” This suggests students followed the guidance in the intervention by questioning whether the study supports the causal claim as unique, and generated their own alternative causal theories.

### Alternative theories

**Fig. 3:**
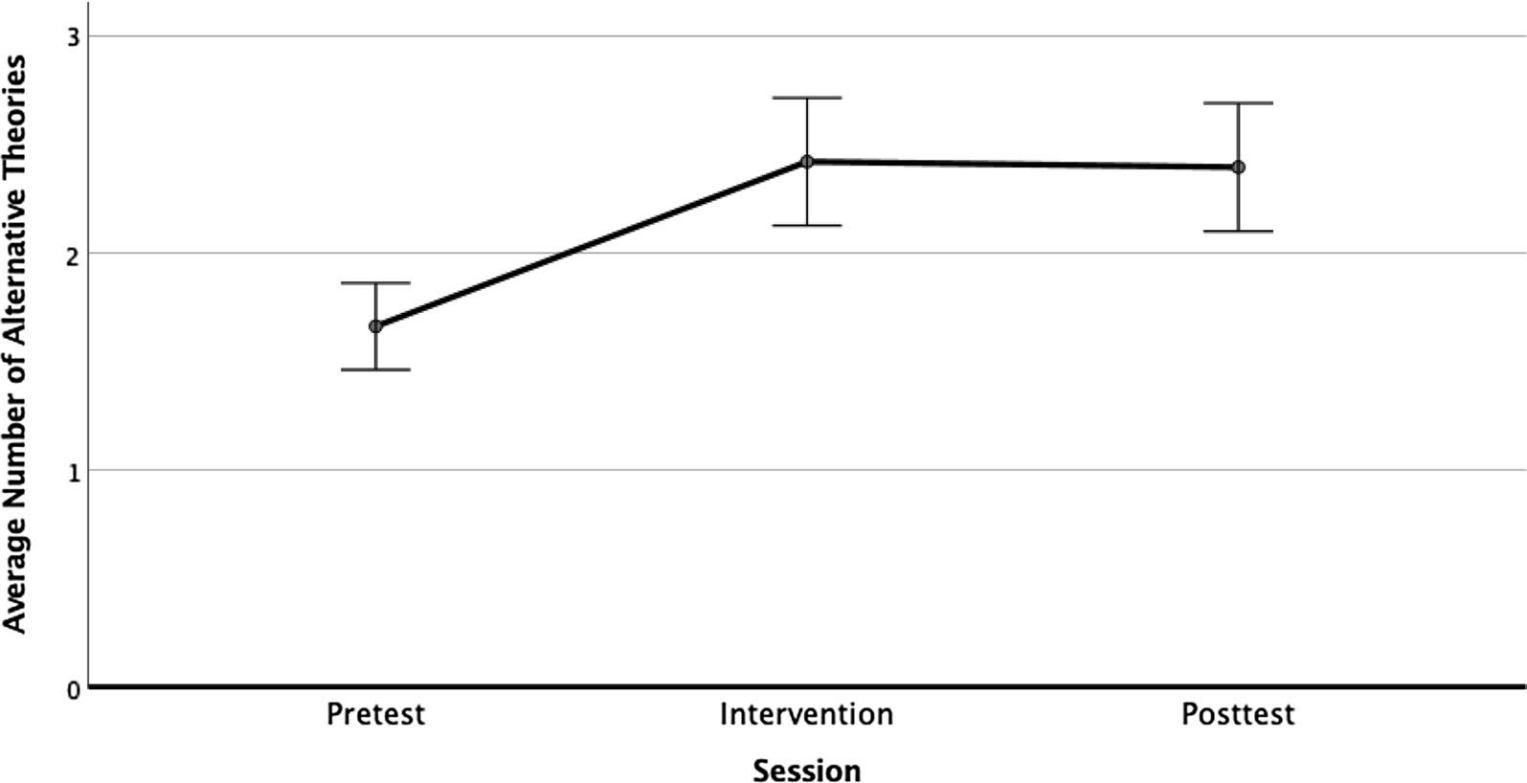
Average number of alternative causal theories generated by students across sessions. Error bars represent within-subjects standard error of the mean (Cousineau, 2005)

In the Diagram task, students created their own representations of possible alternative causal relationships for a causal claim. These causal theories were counted by category, including: (a) direct causal chain, (b) reverse direction chain, (c) common cause (a third variable causes both), and (d) multiple-step chains (intervening causal steps), with scores ranging from 0 to 4 (the same diagram with different variables did not increase the score). A planned linear contrast showed a significant increase in the number of alternative theories included in students’ diagrams (*F*(1,93) = 30.935, *p* < 0.001, *η*_p_^2^ = 0.25) from pre-test (*M* = 1.68, SD = 0.985) to intervention (*M* = 2.44, SD = 1.429), and the gain was maintained at post-test (*M* = 2.40, SD = 1.469) (see Fig. 3). The pretest mean was significantly different (by Tukey’s *LSD*) from both the intervention and the post-test mean (both *p’s* < 0.001), but the intervention and post-test means were not significantly different, *p* = 0.861. Students provided more (and different) theories about potential causes immediately after the intervention, and this improvement was maintained on the post-test a week later.

**Fig. 4:**
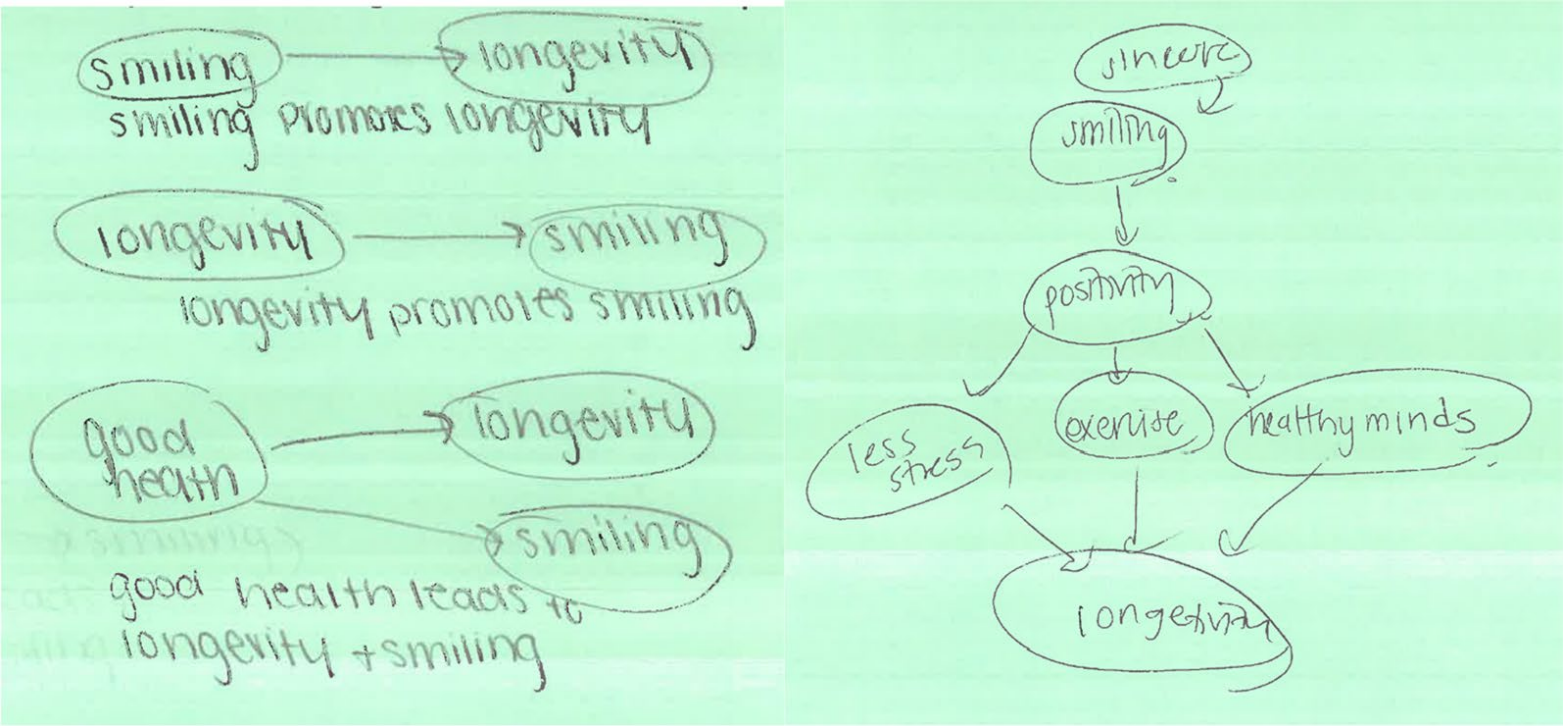
Example diagrams depicting alternative causal theories from a student in the post-test session. On the left, diagrams showing direct cause, reverse cause, and third-variable theories; on right, a more complex theory with multiple steps and paths

On the Diagram task, students increased the number of different alternative theories generated after the intervention, including direct cause, reversed cause-chain, third variables causing both (common cause), and multiple causal steps. This may reflect an increased ability to consider alternative forms of causal theories to the presented causal theory. Students also maintained this gain of 43% in generating alternatives on the post-test measure one week later. Most tellingly, while 34% offered just one alternative theory on the pre-test, *no* students gave just one alternative following the intervention or at post-test. While many described reverse-direction or third-variable links as alternatives (see Fig. 4, left), some offered more novel, complex causal patterns (Fig. 4, right). This improved ability to generate alternative theories to a presented causal claim suggests students may have acquired a foundational approach for avoiding causal theory errors in the future.

## Discussion

The present study documents students’ improvement in avoiding causal theory error when reasoning about scientific findings following a simple, short intervention. The intervention guided students through an extended example of a causal theory error, and included questions designed to scaffold students' thinking about whether a causal claim uniquely accounts for an observed association. Immediately following the intervention and in a delayed test, students increased their reports of problems with causal claims, and generated more alternative causal theories. While causal theory errors have been documented in multiple-choice measures (Bleske-Rechek et al., 2018; Mueller & Coon, 2013; Xiong et al., 2020), this study examined evidence of causal theory errors in students’ open-ended responses to claims in science media reports. This is the first documented intervention associated with significant improvement in avoiding causal theory error from science reports.

### Qualities of successful interventions

The intervention was designed following recommendations from example-based instruction (Shafto et al., 2014; Siegler & Chen, 2008; Van Gog & Rummel, 2010). Worked examples may be especially important in learning to reason about new problems (Renkl et al., 1998), along with explicitly stating the causal theory error and explanation within the example (Große & Renkl, 2007; Stark et al., 2011). Our intervention also emphasized recognizing causal theory errors in *others*’ reasoning, which may be easier to learn than attempting to correct one’s own errors. Finally, introducing diagramming as a means for visually representing causal theories (Ainsworth & Loizou, 2003; Bobek & Tversky, 2016; Gobert & Clement, 1999) may have been helpful in generating alternative theories by using real-world knowledge to “go beyond the information given” (Bruner, 1957; Waldmann et al., 2006). To facilitate this thinking, the extended example was selected for relevance to students based in their own experiences (e.g., interest in math driving their course selections), found to be important in other studies (Bleske-Rechek et al., 2015; Michal et al., 2021). The success of the intervention may be facilitated by an example of causal theory error that “hit close to home” for students through relevance, personal experience, and prior beliefs and attitudes.

In particular, our intervention emphasizing alternative causal theories may assist students in learning to reason about causal claims from correlational studies. Considering multiple causal theories requires original thinking because students must posit additional variables (e.g., third variables not stated in the problem) and unstated causal relationships (e.g., reversed causes, multiple causes and effects). When successful, their alternative theories may help to raise doubt about the internal validity of the study because  the presented causal theory is not unique. The findings show that after the intervention, students provided more of their own competing theories, confirming the existence of alternative causes consistent with the correlational evidence. In reasoning about causes and associations, the process of theory-evidence coordination appears to require less attention to evidence and more attention to alternative theories. In the context of evaluating summaries of science reports (such as those frequently found in the media), considering theory-evidence correspondence cannot disconfirm causal claims; instead, reasoning about other causal theories consistent with the evidence may help to identify when a causal theory is not unique, avoiding causal theory error.

The intervention was immediately followed by decrease in causal theory error appearing in students’ study critiques and alternative theories. However, almost half of the students still rated studies with correlational evidence as “high quality” and “supporting a causal claim” immediately after the intervention even while raising issues with the claim in their written reasons for their ratings. This suggests students may interpret “study quality” and “support for claim” ratings questions by assessing the *consistency* of the correlational evidence and causal theory, while also recognizing that the study cannot *uniquely* account for the finding without  experimental methodology. This suggests open-ended questions may provide information about causal reasoning processes not evident from ratings of support or endorsement of the causal claim alone, as in multiple choice measures (Shou & Smithson, 2015).

In prior work on causal reasoning, people viewed a series of presented data points  to arrive at a causal theory through a process of theory-evidence coordination (Kuhn, 1993; Kuhn et al., 1995), and use varied strategies to determine whether evidence aligns with theories (Kuhn & Dean, 2004; Kuhn et al., 1995). But when summary findings from a study are presented (as in the present study; see also Adams et al., 2017; Bleske-Rechek et al., 2015; Xiong et al., 2020), it is not possible to examine inconsistencies in presented evidence to test alternative theories; instead, the theory-evidence coordination process may focus on addressing whether a presented theory *uniquely* accounts for the observed association. Further studies are needed to better understand how to support students in considering correlational findings, and how people reason about theory and evidence from study summaries in science communications.

### Effective science learning interventions

These results are consistent with prior studies showing that the use of visualizations can improve science learning (Mayer, 2005, 2020). As noted by Gobert and Clement (1999), creating diagrams from scientific material has been linked with better understanding of causation and dynamics, and drawing has been shown to assist learners in some circumstances (Fiorella & Zhang, 2018). In the diagram generation task in our study, students were asked to create their own drawings of possible causal relationships as alternatives to a presented causal claim. Considering their own models  of alternative causal theories in the intervention appeared to have a positive effect on students’ later reasoning. Self-generated explanations supported by visual representations have been shown to improve learners’ understanding of scientific data (Ainsworth & Loizou, 2003; Bobek & Tversky, 2016; Gobert & Clement, 1999; Rhodes et al., 2014; Tal & Wansink, 2016). Previous studies on causal theory error have employed close-ended tasks (Adams et al., 2017; Bleske-Rocher et al., 2018; Xiong et al., 2020), but our findings suggest structuring the learning tasks to allow students to bring their own examples to bear may be especially impactful (Chi et al., 1994; Pressley et al., 1992).

Studies of example-based instruction (Shafto et al., 2014; Van Gog & Rummel, 2010) show improvement in learning through spontaneous, prompted, and trained self-explanations of examples (Ainsworth & Loizou, 2003; Chi, 2000; Chi et al., 1989). In particular, the present study supports the importance of examples to illustrate errors in scientific reasoning. Learning from examples of error has been successful in other studies (Durkin & Rittle-Johnson, 2012; Ohlsson, 1996; Seifert & Hutchins, 1992; Siegler & Chen, 2008), and instructing students on recognizing common errors in reasoning about science may hold promise (Stadtler et al., 2013). Because students may struggle to recognize errors in their own thinking, it may be helpful to provide experience through exposure to others’ errors to build recognition skills. There is some suggestion that science students are better able to process “hedges” in study claims, where less direct wording (such as “associated” and “predicts”) resulted in lower causality ratings compared to direct (“makes”) claims (Adams et al., 2017; Durik et al., 2008). Learning about hedges and qualifiers used in science writing may help students understand the probabilistic nature of correlational evidence (Butler, 1990; Durik et al., 2008; Horn, 2001; Hyland, 1998; Jensen, 2008; Skelton, 1988).

These results are also consistent with theories of cognitive processes in science education showing reasoning is often motivated by familiar content and existing beliefs that may influence scientific thinking (Billman et al., 1992; Fugelsang & Thompson, 2000, 2003; Koehler, 1993; Kuhn et al., 1995; Wright & Murphy, 1984). In this study, when asked to explain their reasons for why the study is convincing, students also invoked heuristic thinking in responses, such as, “…because that’s what I’ve heard before,” or “…because that makes sense to me.” As a quick alternative to careful, deliberate reasoning (Kahneman, 2011), students may gloss over the need to consider unstated alternative relationships among the variables, leading to errors in reasoning (Shah et al., 2017). Furthermore, analytic thinking approaches like causal reasoning require substantial effort due to limited cognitive resources (Shah et al., 2017); so, students may take a heuristic approach when considering evidence and favor it when it fits with previous beliefs (Michal et al., 2021; Shah et al., 2017). Some studies suggest analytic thinking may be fostered by considering information conflicting with beliefs (Evans & Curtis-Holmes, 2005; Evans, 2003a, b; Klaczynski, 2000; Koehler, 1993; Kunda, 1990; Nickerson, 1998; Sá et al., 1999; Sinatra et al., 2014).

### Limitations and future research

Our study examined understanding causal claims from scientific studies within a classroom setting, providing a foundation for students’ later spontaneous causal reasoning in external settings. However, application of these skills outside of the classroom may be less successful. For students, connecting classroom learning about causal theory errors to causal claims arising unexpectedly in other sources is likely more challenging (Hatfield et al., 2006). There is evidence that students can make use of statistical reasoning skills gained in class when approached through an unexpected encounter in another context (Fong et al., 1986), but more evidence is needed to determine whether the benefits of this intervention extend to social media reports.

As in Mueller and Coon’s (2013) pre/post classroom design, our study provided all students with the intervention by pedagogical design. While the study took place early in the term, it is possible that students showed improvement over the sessions due to other course activities (though correlational versus experimental design was not yet addressed). Additional experimental studies are needed to rule out possible external influences due to the course instruction; for example, students may, over the two-week span of the study, have become more skeptical about claims from studies given their progress in learning about research methods. As a consequence, students may have become critical of all studies (including those with experimental evidence) rather than identifying concerns specific to claims from correlational studies. In addition, the advanced psychology students in this study may have prior knowledge about science experiments and more readily understand the intervention materials, but other learners may find them less convincing or more challenging. Psychology students may also experience training distinguishing them from other science students, such as an emphasis on critical thinking (Halpern, 1998), research methods (Picardi & Masick, 2013), and integrating knowledge from multiple theories (Green & Hood, 2013; Morling, 2014). They may also hold different epistemological beliefs, such as that knowledge is less certain and more changing (Hofer, 2000) or more subjective in nature (Renken et al., 2015). Future research with more diverse samples at varying levels of science education may suggest how novice learners may benefit from instruction on causal theory error and how it may impact academic outcomes.

The longitudinal design of this classroom study extended across 2 weeks and resulted in high attrition; with attendance optional, only 40% of the enrolled students attended all three sessions and were included in the study. However, this subsample scored similarly (*M* = 146.25, SD = 13.4) to nonparticipants (*M* = 143.2, SD = 13.7) on final course scores, *t*(238) = 1.16, *p = *.123. Non-graded participation may also have limited students’ motivation during the study; for example, the raw number of diagrams generated dropped after the first session (though quality increased in later sessions). Consequently, the findings may underestimate the impact of the intervention when administered as part of a curriculum. Further studies are also needed to determine the impact of specific intervention features, consider alternative types of evidence (such as experimental studies), and examine the qualities of examples that motivate students to create alternative theories.

Finally, the present study examines only science reports of survey studies with just two variables and a presumed causal link, as commonly observed in media reports focusing on just 1 predictor and 1 outcome variable (Mueller, 2020). For both science students and the public, understanding more complex causal models arising from varied evidence will likely require even more support for assessment of causal claims (Grotzer & Shane Tutwiler, 2014). While the qualities of evidence required to support true causal claims is clear (Stanovich, 2009, 2010), causal claims in non-experimental studies are increasingly widespread in the science literature (Baron & Kenny, 1986; Brown et al., 2013; Kida, 2006; Morling, 2014; Reinhart et al., 2013; Schellenberg, 2020). Bleske-Rechek et al. (2018) found that half of psychology journal articles offered unwarranted direct causal claims from non-experimental evidence, with similar findings in health studies (Lazarus et al., 2015). That causal claims from associational evidence appear in peer-reviewed journals (Bleske-Rechek et al., 2018; Brown et al., 2013; Lazarus et al., 2015; Marinescu et al., 2018) suggests a new characterization is needed that acknowledges how varied forms of evidence are used in scientific arguments for causal claims. As Hammond and Horn (1954) noted about cigarette smoking and death, accumulated associational evidence in the absence of alternative causal theories may justify the assumption of a causal relationship even in the absence of a true experiment.

### Implications

Learning to assess the quality of scientific studies is a challenging agenda for science education (Bromme & Goldman, 2014). Science reports in the media appear quite different than those in journals, and often include limited information to help the reader recognize the study design as correlational or experimental, or to detect features such as random assignment to groups (Adams et al., 2017; Morling, 2014). Studies described in media reports often highlight a single causal claim and ignore alternatives even when identified explicitly in the associated journal article (Adams et al., 2019; Mueller & Coon, 2013; Sumner et al., 2014). Reasoning “in a vacuum” is often required given partial information in science summaries, and reasoning about the potential meaning behind observed associations is a key skill to gain from science education. In fact, extensive training in scientific methods (e.g., random assignment, experiment, randomized controlled trials) may not be as critical as acknowledging the human tendency to see a cause behind associated events (Ahn et al., 1995; Johnson & Seifert, 1994; Sloman, 2005). Consequently, causal theory errors are ubiquitous in many diverse settings where underlying causes are unknown, and learning to use more caution in drawing causal conclusions in general is warranted.

However, leaving the task of correcting causal theory error to the learner falls short; instead, both science and media communications need to provide more accurate information (Adams et al., 2017, 2019; Baram-Tsabari & Osborne, 2015; Bott et al., 2019; Yavchitz et al., 2012). Our findings suggest that science media reports should include both alternative causal theories and explicit warnings about causal theory error. More cautious claims and explicit caveats about associations provide greater clarity to media reports without harming interest or uptake of information (Adams, et al., 2019; Bott et al., 2019), while exaggerated causal claims in journal articles has been linked to exaggeration in media reports (Sumner et al., 2014). For example, press releases about health science studies aligning claims (in the headline and text) with the type of evidence presented (correlational vs. randomized controlled trial) resulted in later media articles with more cautious headlines and claims (Adams et al., 2019). Hedges and qualifiers are frequently used in academic writing to accurately capture the probabilistic nature of conclusions and are evaluated positively in these contexts (Horn, 2001; Hyland, 1998; Jensen, 2008; Skelton, 1988). For example, Durik and colleagues (2008) found that hedges qualifying interpretative statements did not lead to more negative perceptions of research. Prior work has demonstrated that headlines alone can introduce misinformation (Dor, 2003; Ecker et al., 2014a, b; Lewandowsky et al., 2012), so qualifiers must appear with causal claims.

The implications of our study for teaching about causal theory error are particularly important for psychology and other social, behavioral, and health sciences where associational evidence is frequently encountered (Adams et al., 2019; Morling, 2014). Science findings are used to advance recommendations for behavior and public policies in a wide variety of areas (Adams et al., 2019). While many people believe they understand science experiments, the point of true  experiments—to identify a cause for a given effect—may not be sufficiently prominent in science education (Durant, 1993). Addressing science literacy may require changing the focus toward recognizing causal theory error rather than creating expectations that all causal claims can be documented through experimental science. Because people see associations all around them (Sloman, 2005; Sloman & Lagnado, 2015), it is critical that science reports acknowledge that their presented findings stop far short of a "gold standard" experiment (Adams et al., 2019; Sumner et al., 2014; Hatfield et al., 2006; Koch & Wüstemann, 2014; Reis & Judd, 2000; Sullivan, 2011). Understanding the low base rate of true experiments in science and the challenges in establishing causal relationships is key to appreciating the value of the scientific enterprise. Science education must aim to create science consumers able to engage in *argument* from evidence (NTSA Framework, 2012, p. 73), recognizing that interpretation is required even with true experiments through evaluating internal and external validity. By encouraging people to consider the meaning of scientific evidence in the world, they may be more likely to recognize causal theory errors in everyday life. The present study provides some evidence for a small step in this direction.

## Conclusions

The tendency to infer causation from correlation—referred to here as *causal theory error*—is arguably the most ubiquitous and wide-ranging error found in science literature, classrooms, and media reports. Evaluating a probabilistic association from a science study requires a different form of theory-evidence coordination than in other causal reasoning tasks; in particular, evaluating a presented causal claim from a correlational study requires assessing the plausibility of alternative causal theories also consistent with the evidence. This study provides evidence that college students commit frequent causal theory error in interpreting science reports as captured in open-ended reasoning about the validity of a presented causal claim. This is the first identified intervention associated with significant, substantial changes in students’ ability to avoid causal theory error from claims in science reports. Because science communications are increasingly available in media reports, helping people improve their ability to assess whether studies support potential changes in behavior, thinking, and policies is an important direction for cognitive research and science education.

## Data Availability

All data and materials are available upon reasonable request.

## Appendix 1

**Intervention in session 2**

(Labels shown in italics did not appear in participants’ materials.)

Please read the following packet and answer the questions to the best of your ability as you go along.

**Algebra 2 and career success**

In 2006, researchers at Educational Testing Service (ETS) conducted an experiment in which they followed students for 12 years starting in 8th grade. They found that 84% of the top-tier workers (receiving the highest pay) had taken Algebra 2 classes in high school. In contrast, only 50% of those in the lowest pay tier had taken Algebra 2. This result suggests that requiring students to take Algebra 2 would benefit all students and can prepare them for better jobs after high school. Twenty states, including Michigan, have passed laws making Algebra 2 a graduation requirement for all of their high school students.

**1. *****Evidence quality*** Does the ETS study convince you that taking Algebra 2 would be beneficial for students? Why or why not?

**2. *****Causal theory error*** Do you think that requiring Algebra 2 based on the ETS study was a good decision for the state? Why or why not?

The Algebra 2 requirement has now fallen out of favor in some states, and they are now reversing or watering down their Algebra 2 requirement. In Michigan, students can now take a “Career Technical Education” course instead of Algebra 2.

**3. *****Counterfactual*** How do you think that *no* required Algebra 2 course will affect Michigan students’ career opportunities?

Some people (and legislators) view the ETS study as proving that taking Algebra 2 causes students to have better chances of getting top-tier jobs. That certainly seems plausible because having math skills should help you get a good job!

**4. *****Causal mechanism*** Why might taking Algebra 2 lead to students getting a better job?

However, it is not necessarily true that taking Algebra 2 would lead directly to getting better jobs. It is possible that the connection between Algebra 2 and higher job status is that the students who took Algebra 2 differed in other ways from people who did not.

**5. *****Equality of groups*** In what ways might students in Algebra 2 differ from students who don’t take it? Who chooses to take Algebra 2? Try to think of other differences that might lead some students toward top-tier jobs. List at least 2 different things.

**6. *****Self-selection*** Think back to the ETS study, when students had to choose whether to take Algebra 2. Why would a high school student decide to take it, and why would a student decide not to take it? Try to think of at least one new reason a student would take it, and one reason not to take it.

**7. *****Third variables*** Think about the reasons listed below, and judge whether each reason might also explain why algebra students end up in better jobs. Mark each sentence with a “T” for true and “F” for false based on whether you think it is a good reason. **A:** Students who chose Algebra 2 were also smarter, so they did well in school and got better jobs. **B:** Students who chose Algebra 2 were also going to college, so they ended up in better jobs. **C:** Students who chose Algebra 2 were from richer families who help them end up in better jobs. **D:** Students who chose Algebra 2 went to better high schools (with more math classes), and therefore they ended up in better jobs.

From the previous questions, notice that there are other reasons that students might both have taken Algebra 2 and gotten a top-tier job. It might look like what the students learned in Algebra 2 helped them get good jobs, but it could have been one of these other reasons that was the real cause.

***Description of causal theory error ***The Michigan legislators seem to make a mistake in their decision making (as all people sometimes do) called the “correlation-to-causation” error. Just because two things are related (like Algebra 2 and better jobs), you can’t decide that one causes the other. Taking Algebra 2 and getting a good job may both be caused by something else; for example, being good in school, being in a good school, or having parents that are doctors. This is called a “third variable” explanation, where two things “go together” because of some other (third) cause.

Take a moment to pause and think: Most of us already know that “correlation does not imply causation;” but many times, especially when the cause makes sense, we forget to question our assumptions and make a **causal theory error**.

If any two variables A and B are related, it can mean one of several things:

- It could mean that A causes B.
- It could mean that B causes A.
- It could mean that C causes both A and B. Or, A and B both can cause C.

It could be really complicated, in which A causes B which causes more A, and so on. Or, that A causes B, but C also impacts B. Whenever you evaluate evidence, it is important to think through these various possibilities.

In this next exercise, you will have a chance to evaluate evidence like the ETS study about Algebra 2. You will be asked to draw simple pictures to show possible reasons why two variables like *Algebra 2* and *job quality* are related.

**8. *****Correcting causal theory error*** Does this model make sense? Y or N

You already wrote down why Algebra 2 might help people get good jobs; for example, good jobs may require math skills.

Now consider:

**9. *****Direction of causation*** Does this model make sense? Y or N

Probably not, because taking Algebra 2 usually happens before graduating and getting a job. So that is not a likely cause.

***10. Identify third variable*** Does this model make sense? Y or N

It’s a good one… But you might have an even more complicated model in mind, like this next one:

Clearly, there are many other causes that may help you end up in a top-tier job. So how do you decide which ones to choose?

***Complex causal models*** How do scientists decide which is the right model? They tend to use other information, like variables they know are potentially relevant, clear causal explanations, other known relationships, and so forth. In other words, a simple correlation is not enough information to know for sure, but when combined with more information, you may be able to piece together a convincing explanation. But this takes some work on your part, so be ready to slow down and think when you consider evidence.

***Advice*** When you read a media article about a scientific study, you may not have all the information you need to evaluate a causal model. But when you’re reading about a causal model, be extra careful if the cause seems to “make sense:” That is a good time to question yourself about other possible causes for the observed evidence. Ready to try this right now?

## Appendix 2

### Task materials

#### Silence or music

Although some people prefer to work in silence, many people opt to listen to music while working or studying. In fact, a brief glimpse into a library or coffee shop will reveal dozens of individuals poring over their laptops and books with earphones wedged into their ears. Researchers have recently become interested in whether listening to music actually helps students pay attention and learn new information. A recent study was conducted by researchers at a large midwestern university. Students (*n* = 450) were surveyed prior to final exams in several large, lecture-based courses, and asked whether or not they had listened to music while studying for the final. The students who listened to music had, on average, higher test scores than students who did not listen to music while studying. The research team concluded that students who want to do well on their exams should listen to music while studying.

#### Computers-glasses

A recent study funded by the National Institute of Health links extended computer use to an increase in glasses and contacts use. Researchers recruited 600 employees from the NYC area and administered a survey on computer use. Of the respondents who used the computer for 30+ h a week, 2/3 wore corrective lenses or contacts and had severe myopia (nearsightedness) or hyperopia (farsightedness). In contrast, people who did not use computers extensively at work were less likely to report wearing corrective lenses: only 10% of people who used computers less than 30 h a week at their jobs required corrective lenses. To avoid harming your eyes, the researchers recommend avoiding too many hours using a computer each day. If it is impossible to avoid screen time on the job, they recommend speaking with an ophthalmologist about what you might do to counteract the negative consequences of screen time.

#### Parent-self control

An important aspect of parenting is to help children develop their self-control skills. Developmental scientists have long been interested in how parenting practices impact children’s ability to make their own choices. As part of a recent study, researchers measured children's body fat and surveyed mothers about the amount of control they exert over their children's eating. The results of this study, conducted with 400 children aged 3 to 5, found that those with the most body fat had the most “controlling” mothers when it came to the amount of food eaten. This shows that, “when mothers exert more control over their children's eating, the children display less self-control,” researchers said. Researchers recommend that parents should avoid being too controlling, and let their children learn to develop their own skills.

#### Church-health

Now, please diagram possible relationships between variables suggested by this news headline: “Church attendance boosts immunity.” Be sure to label the circles and links in your diagrams.

Then, write a caption below each diagram to describe the relationships.

#### Smiling-longevity

Now, please diagram possible relationships between variables suggested by this news headline: “Sincere smiling promotes longevity.” Be sure to label the circles and links in your diagrams.

Then, write a caption below each diagram to describe the relationships.

#### Breast-feeding and Intelligence

Now, please diagram possible relationships between variables suggested by this news headline: “Breast-fed children found smarter.” Be sure to label the circles and links in your diagrams.

Then, write a caption below each diagram to describe the relationships.

## Appendix 3

### Study critique task

#### Silence or music

1. Please rate the quality of the study described above.

**Table:**

*Low quality*	1	2	3	4	5	*High quality*

2. To what extent do you think that the study supports the conclusion that one should listen to music while studying?

**Table:**

*Not at all*	1	2	3	4	5	*Very much*

3. Please write your critical evaluation of the study and its conclusions: What is good, and bad, about this news report?

Example response showing causal theory error:

- “Good: Studies different groups, reaches a reasonable conclusion based off of the analysed data. Bad: Makes no reference to the type of food the mothers were controlling the children to eat, etc.”

Example response avoiding causal theory error:

- “The study was conducted well for a correlation finding but not for causation. Therefore, the conclusions the news report and study made cannot be supported.”

## Appendix 4

### Causal diagram task

#### Diagramming task and example student responses:

Now, please diagram possible relationships between variables suggested by this news headline: “Breast-fed children found smarter.” Be sure to label the circles and links in your diagrams.

Then, write a caption below each diagram to describe the relationships.
